# A Randomized Controlled Trial Examining the Effects of Mindful Eating and Eating without Distractions on Food Intake over a Three-Day Period

**DOI:** 10.3390/nu14051043

**Published:** 2022-02-28

**Authors:** Lana Seguias, Katy Tapper

**Affiliations:** Department of Psychology, School of Arts and Social Sciences, City, University of London, London EC1R 0JD, UK; lana.seguias@city.ac.uk

**Keywords:** mindful eating, attentive eating, mindfulness, distraction, diet, weight management

## Abstract

This study compared the effects of mindful eating and eating without distractions on energy intake and diet over a 3-day period among healthy-weight females. Mindful eating was defined as attending to the sensory properties of one’s food as one eats. Participants (*n* = 99) were asked to either focus on the sensory properties of their food (MIND), eat without distractions (CON-D) or they were not provided with any instructions (CON-I). All participants completed an online food recall measure at the end of each day. Those in the MIND and CON-D groups also rated strategy adherence at the end of each day. Results showed no significant effects of condition on energy intake (*η_p_*^2^ = 0.00), saturated fat, added sugar and fiber (*η_p_*^2^ = 0.03), or fruit and vegetables (*η_p_*^2^ = 0.04). There was also no significant relationship between energy intake and strategy adherence in the MIND group (*r* = −0.02). For those in the CON-D group, there was a trend toward a negative relationship between energy intake and strategy adherence (*r* = −0.31, *p* = 0.085). Among this population, there was no evidence that asking people to attend to the sensory properties of their food improved their diet. Further research is needed to identify mechanisms underpinning significant effects observed in laboratory studies, to help understand when this strategy is, and is not, likely to be helpful.

## 1. Introduction

Mindfulness is increasingly being promoted as an effective way of eating more healthily or of managing one’s weight. However, whilst existing mindful eating interventions tend to utilize comprehensive training in both mindfulness and mindful eating (e.g., MB-EAT [1]), rising levels of obesity, and associated treatment costs, means there is also a need for less resource-intense, evidence-based strategies that can be delivered as simple advice. Such advice could feed into public health interventions aimed at promoting healthy eating as well as interventions designed to aid weight loss maintenance or weight gain prevention, where small reductions in energy intake over time may help counter a general tendency for people to gain weight. In such cases, the population may or may not suffer from being overweight and will not necessarily be actively trying to lose weight. Thus, an important question is whether advice to use a simple mindful eating strategy can act as a “nudge” that reduces food intake and/or promotes healthy eating without the individual necessarily engaging in effortful goal striving. 

One potential candidate for such a strategy is paying attention to the sensory properties of food whilst eating [2]. A number of experimental studies conducted in laboratory settings have found that use of this strategy (in the absence of any additional training) can reduce intake of high-calorie foods [3,4,5,6,7,8]. However, other studies have failed to replicate this effect [5,9,10,11]. A related strategy is that of minimizing distractions whilst eating, for example, by turning off the television or stopping work, since distractions have been shown to increase intake, as well as levels of hunger and subsequent eating [12,13,14,15]. This raises the question of whether (a) advising people to pay attention to the sensory properties of their food can reduce food intake outside of a laboratory setting and (b) whether this strategy is more effective than advising people to eat without distractions.

The main aim of the present study was therefore to compare the effects of these two different strategies with a control group who were not asked to alter the way they ate. The study looked at effects on the amount of food eaten (in terms of energy) as well as indications that people might be making healthier choices (in terms of intake of saturated fats, added sugars, fiber and fruit/vegetables). The strategies were implemented outside the laboratory over a three-day period. Since most of the significant effects in the laboratory have been obtained with samples that are predominantly female and of a healthy weight, the current study recruited a similar demographic. Although this may limit the immediate applicability of the findings to weight loss interventions, it allows for a more direct comparison with the body of laboratory research. 

The present study also collected participant reports on the extent to which they used the mindful eating/no distraction strategy assigned to them. This allowed for further tests of the relationship between these strategies and food intake. Additionally, the study explored the notion that mindful eating might give people more pleasure [2,16] as well as the possibility that reductions in serving sizes might be compensated for by increased frequency of eating [2].

## 2. Materials and Methods

### 2.1. Participants

Participants were females (*n* = 99) with an average age of 22.4 years (SD = 5.0) and an average BMI of 22.3 (SD = 1.4), recruited via an advertisement placed on an online platform affiliated with the university, as well as via flyers handed out to individuals and placed on billboards around the university. Participants were told the study was about “eating behaviours” but, to limit halo effects and demand characteristics, they were not made aware of the specific hypotheses and no reference was made to mindfulness or mindful eating. Participants who completed the study received 20 pounds sterling. Inclusion criteria were female, aged 18 or over, fluent English speaker, a self-reported BMI of between 20 and 25 and having access to a mobile phone and to the internet during the evenings. Exclusion criteria were dieting to lose weight, taking medication that affected their appetite, a current or previous diagnosis of an eating disorder, or participation in a related study. Ethical approval was granted by City, University of London Psychology Department Research Ethics Committee.

### 2.2. Study Design, Randomization and Outcome Measures

The study employed a three-armed, parallel groups, single-blind randomized controlled trial design in which participants were allocated equally to a mindful eating condition (MIND), a no distraction control (CON-D) or a no instruction control (CON-I). KT used the website random.org to randomize the order of the 99 trials, though there was no researcher blinding or allocation concealment. The primary outcome was total calorie intake over a 3-day period. Secondary outcomes were total intake of non-milk extrinsic sugars, saturated fat, fruit and vegetables and fibre, measured in grams. To aid interpretation, all outcomes have been reported as means per day rather than total intakes. The study was registered at clinicaltrials.gov, accessed on 28 December 2021 (NCT03601650).

### 2.3. Smartphone Application and Daily Messages

A mobile phone application sent participants reminders and provided access to an audio recording. Every evening, over a 3-day period, all participants were sent a notification reminding them to complete an online food recall measure. Participants in the MIND and CON-D groups also had access to an audio recording that they could listen to at any point over the three days. In the MIND group. this audio was based on the Raisin Meditation [17] and encouraged participants to focus on the sensory properties of their food, i.e., its smell, look, taste, texture, temperature and the physical acts of chewing and swallowing. In the CON-D group, the audio encouraged participants to avoid distractions whilst eating. The length of these recordings were 1 min and 57 s and 50 s, respectively. Both ended by reminding participants to listen to the audio just before eating. (See Supplementary materials for full scripts.) The number of times the clip was played in full was recorded by the application. 

Daily messages were provided to MIND and CON-D participants in three different envelopes labelled Tuesday, Wednesday, and Thursday. In each of these envelopes, there were four other envelopes labelled with the day of the week and “Please open before breakfast”, “Please open before lunch”, “Please open before dinner” and “Food Diary. Please open at 8:30 p.m.” The messages inside the first three envelopes provided prompts to encourage participants to attend to the sensory properties of their food (MIND) or to eat without distractions (CON-D) (see Supplementary Materials). The message in the fourth envelope asked participants to respond to two questions: (a) how much they had focused on the sensory properties of their food whilst eating (MIND)/avoided distractions whilst eating (CON-D) and (b) how well they thought they had remembered the food they had eaten that day. Both questions were rated on a scale of 1 to 5, anchored by “not at all” and “very much”. The message also reminded participants to complete the food recall measure and provided them with the website link as well as their username and password. Additionally, participants were asked to bring their responses with them to their second laboratory appointment.

Participants in the CON-I group also received envelopes labelled with the days of the week but these only contained the Food Diary envelope that included the food recall reminder and question (b) relating to memory (i.e., it did not include the question (a) relating to the extent to which they had attended to the sensory properties of their food/eaten without distractions).

### 2.4. Food Recall Measure

Primary and secondary outcomes were assessed using Intake24, a computerized, multiple pass, 24-h dietary recall measure with a database of over 2500 foods and portion sizes images (www.intake24.co.uk, accessed on 28 December 2021). The software has been validated against interviewer-led 24-h recalls with 17–24-year-olds [18] and automatically computes energy intake and intake of saturated fat, added sugar and fiber for the researcher. Fruit and vegetable intake was calculated by summing the weight of all fruit and vegetables consumed. However, since five-a-day guidelines tend to restrict the contribution of juices, smoothies, potatoes and legumes to five-a-day targets, these items were excluded from the calculation of fruits and vegetables. Where dishes were fruit- or vegetable-based (such as vegetable lasagna or apple pie), the weight was recorded as half the total weight of the dish. 

### 2.5. Background and Feedback Questionnaires

At the start of the study, all participants were asked to provide their age and ethnicity (White; Black or Black British; Asian or Asian British; Mixed; Other). At the end of the study, participants in the MIND and CON-D groups completed a feedback questionnaire that asked them to rate how much they liked: (a) receiving the daily messages, (b) listening to the audio recording, and (c) completing the food recall. The rating scale ranged from 1–5, where 1 represented “I didn’t like it at all” and 5 represented “I really liked it.” Participants were also asked to provide reasons for their ratings and to specify whether they had used a mobile phone or computer to complete the food recall measure on each of the three days. The second section of the questionnaire asked participants to indicate whether or not they felt taking part in the study had influenced the amount or type of food they had eaten. Where they answered yes, they were asked to provide an explanation. The third section of the questionnaire asked participants about the pleasure they experienced from the food they ate over the course of the study on a scale of 1–5, where 1 represented “I didn’t get much pleasure from the food I ate” and 5 represented “I got a lot of pleasure from the food I ate.” Participants were also asked to describe the amount of pleasure they got from their food as either: (a) less than usual, (b) about the same as usual, (c) more than usual.

Participants in the CON-I group completed the same feedback questionnaire but without the items relating to the daily messages or audio recordings.

### 2.6. Procedure

Recruitment began in January 2017 and data collection was completed in February 2019 when the target sample size was reached. LS conducted screening by telephone and met with participants at the university on a Monday and a Friday. The main intervention and data collection phase occurred from Tuesday to Thursday. On the Monday, the participant provided demographic information and LS talked them through a demonstration trial of the food recall measure. Participants were advised to complete the recall measure on a computer rather than a mobile phone and were also instructed to keep a record of any foods they forgot to enter. LS then showed participants the messages, demonstrated the mobile application and asked those in the MIND and CON-D groups to try to listen to the audio recording before every meal.

**Figure 1:**
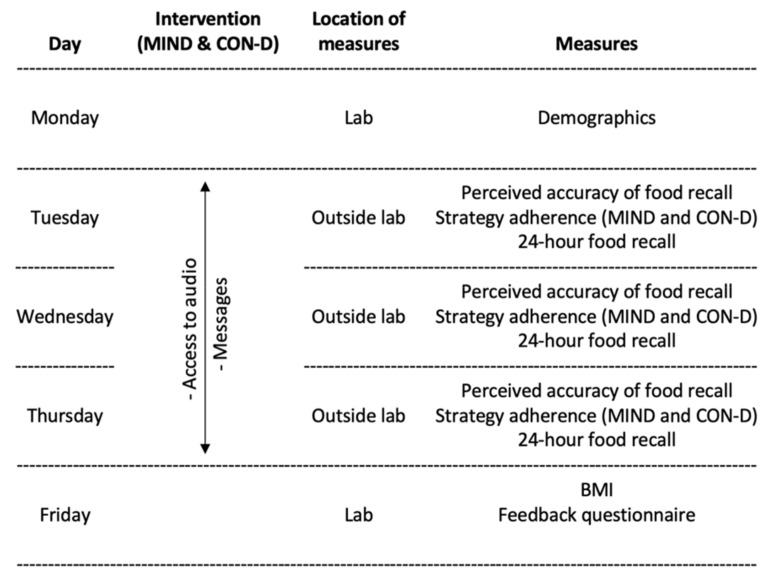
Summary of measures and procedures.

Over the next three days (Tuesday–Thursday), participants read the messages and completed the food recall measure. On Friday, participants returned to the laboratory where LS measured their weight and height, administered the feedback questionnaire and asked about any items participants had forgotten to record. If participants had consumed an average of more than 2500 calories per day or less than 1000 calories per day, LS went through the diary with them to confirm the items listed. Figure 1 provides a summary of the study measures and procedures.

### 2.7. Sample Size and Power

The sample size was informed by both pragmatic issues and by data from Seguias and Tapper [4] that showed a large effect size when comparing mindful eating with a no instruction control. The sample size of 33 per condition allowed for the detection of a difference of 200 kcal per day between groups, assuming an average calorie intake of 2000 kcal (the recommended daily energy intake for women) and a standard deviation of 400.

### 2.8. Statistical Analysis 

**Figure 2:**
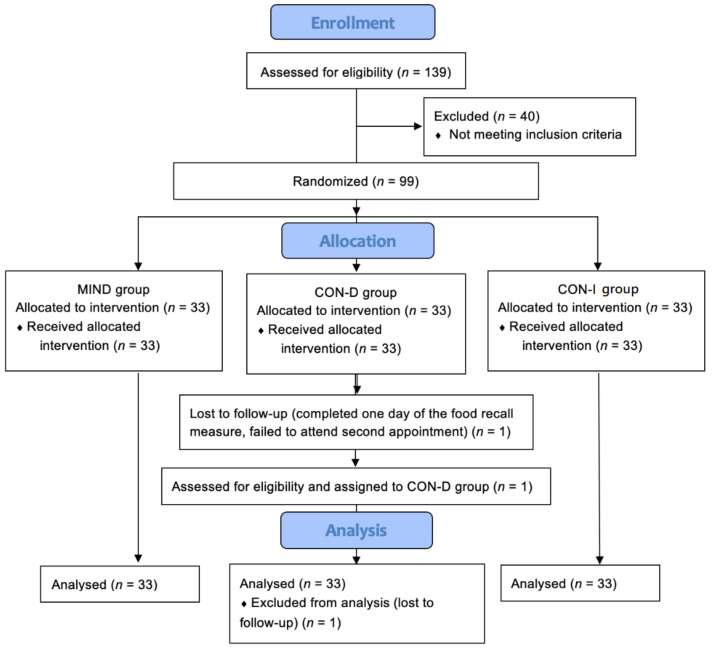
Flow of participants through the study.

Figure 2 shows the flow of participants through the study. Since the study was designed to assess the effects of specific instructions on food intake (as opposed to the effects of a particular intervention), analysis was per-protocol only. All data were analyzed using IBM SPSS Statistics (version 22). 

For the confirmatory analysis, square root transformations were applied to saturated fat, added sugar, fiber and fruit/vegetables to correct for positive skew. The effects of condition on saturated fat, added sugar and fiber were analyzed using a one-way MANOVA. Since energy intake and intake of fruit/vegetables would have correlated with other outcomes, these were analyzed separately using two one-way ANOVAs. 

For the exploratory analysis, a mixed ANOVA was used to analyze the effects of time and condition on the extent to which participants in the MIND and CON-D groups reported using their assigned strategy. Mean overall strategy use was normally distributed in the CON-D group but negatively skewed in the MIND group. Therefore, Pearson’s correlation was used to examine the relationship between overall strategy use and energy intake in the CON-D group, whilst Spearman’s correlations were used to examine relationships between strategy use and untransformed scores for other macronutrients in the CON-D group and for all outcomes in the MIND group. An ANOVA was used to compare group differences in mean ratings of pleasure, and chi-square was used to compare group differences in relative pleasure. The number of eating occasions per day was counted for each participant from the Intake24 data and a mean across the three days was computed. An ANOVA was used to compare group differences in these means. Chi-square was used to compare the proportion of participants in each group who felt the study had influenced the amount and type of food they had eaten.

## 3. Results

### 3.1. Participant Characteristics

As shown in Table 1, participants in the three groups were well matched in terms of both age and BMI and the majority of participants reported using a computer to complete the food recall measure rather than a mobile phone. However, more participants in the MIND group described themselves as Asian, slightly more participants in the CON-D group described themselves as Black and more participants in the CON-I group described themselves as White. 

Participant ratings of how well they thought they had remembered the food they had eaten was similar across conditions, averaging 4.5 (SD = 0.5) in the MIND group, 4.4 (SD = 0.6) in the CON-D group and 4.5 (SD = 0.5) in the CON-I group. 

### 3.2. Effect of Condition on Energy and Macronutrient Intake

**Figure 3:**
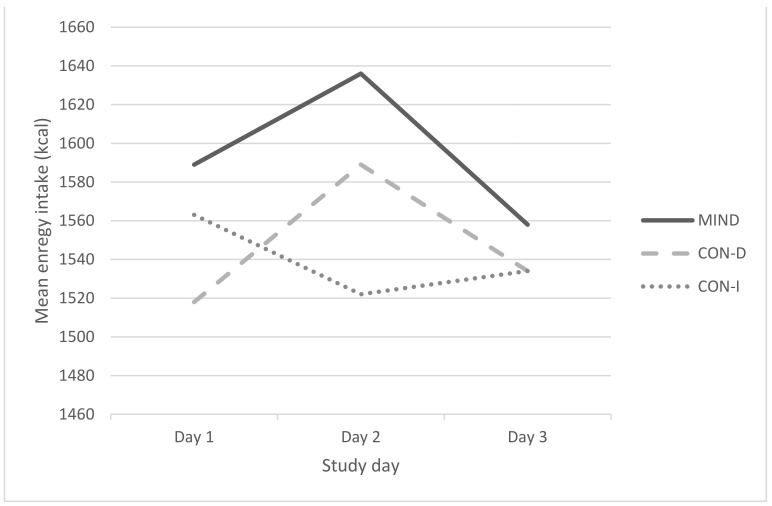
Mean energy intake (kcal) across the 3-day study period for participants in each of the three conditions.

Table 2 shows the mean daily energy and macronutrient intakes across the three conditions, whilst Figure 3 shows the mean daily energy intake across the three conditions broken down by day. There were no significant effects of condition on energy intake, *F*(2, 96) = 0.12, *p =* 0.89, *η_p_*^2^ = 0.002, on intake of saturated fat, added sugar and fiber, *F*(6, 188) = 0.93, *p =* 0.48, *η_p_*^2^ = 0.03, or on intake of fruit and vegetables, *F*(2, 96) = 1.80, *p =* 0.17, *η_p_*^2^ = 0.04. (Repeating these analyses with BMI as a covariate did not alter this pattern of results.) 

### 3.3. Exploratory Analysis of Process Measures: Strategy Use

Over the 3-day period, those in the MIND condition listened to the audio recording between 0 and 18 times (*mdn* = 2), whilst those in the CON-D condition listened to it between 0 and 6 times (*mdn* = 1). Five people in the MIND group and 10 in the CON-D group failed to listen to the recording at all. 

Table 3 shows mean ratings of the extent to which MIND and CON-D participants reported attending to the sensory properties of their food/eating without distraction during the study period. Analysis showed a significant linear effect of time on ratings, *F*(1, 64) = 9.96, *p =* 0.002, *η_p_*^2^ = 0.14, that did not differ by condition, *F*(1, 64) = 1.11, *p =* 0.30, *η_p_*^2^ = 0.02. However, there were no significant relationships between the overall extent to which participants used their assigned strategy and their food intake (Table 4), though the extent to which participants ate without distraction showed a trend toward a negative relationship with energy intake. 

### 3.4. Exploratory Analysis of Additional Outcomes: Pleasure and Eating Occasions

Mean ratings of the amount of pleasure participants experienced from the food they ate were 3.8 (SD = 0.7) in the MIND group, 3.9 (SD = 0.9) in the CON-D group and 3.9 (SD = 0.9) in the CON-I group. There were no significant differences between groups, *F*(2, 96) = 0.36, *p =* 0.70, *η_p_^2^* = 0.01. The categorical measure of relative pleasure showed that, compared to those in the CON-I group, more participants in the CON-D group reported experiencing more pleasure than usual from their food, and an even greater proportion in the MIND group reported experiencing more pleasure than usual from their food (Table 5). These differences showed a trend towards significance, χ(4) = 8.62, *p* = 0.07.

The mean number of eating occasions per day was 4.1 (SD = 0.9) in the MIND group, 4.2 (SD = 0.8) in the CON-D group and 4.4 (SD = 0.9) in the CON-I group. These differences were not significant, *F*(2, 96) = 0.72, *p =* 0.49, *η_p_^2^* = 0.02.

### 3.5. User Views and Comments

Participant ratings of how much they liked receiving the daily messages were 4 (SD = 1) in the MIND group and 4 (SD = 1) in the CON-D groups. Mean ratings of the audio recordings were 3 (SD = 1) in the MIND group and 3 (SD = 1) in the CON-D group. The comments provided by participants showed that whilst some enjoyed the messages and audio and found them helpful, others said they were used to eating with others or doing other things whilst eating (such as watching television, reading, walking or talking on the phone) and that it was hard to adjust these habits or that they did not want to adjust these habits. Some participants commented that the messages and audio were repetitive and that the audio was redundant as it simply repeated the information contained in the messages.

In general, participants liked completing the food recall measure, giving it a mean rating of 4 (SD = 1). Participants commented that it was easy and enjoyable to complete. Many participants commented that it made them more aware of their eating habits. 

Overall, 25% of participants felt the study had influenced the amount of food they had eaten and 20% felt the study had influenced the type of food they had eaten, but there were no significant differences between groups (Table 6; χ(2) = 3.00, *p* = 0.22 and χ(2) = 0.90, *p* = 0.64 for amount and type, respectively). A total of 39 participants provided at least one comment that gave further detail; 16 in the MIND group, 12 in the CON-D group and 11 in the CON-I group. Three participants in the MIND group and 3 in the CON-D group felt they had eaten less or had eaten more healthily because of the strategy. An additional 2 participants in the MIND group reported replacing soup with foods with more texture. However, the majority of comments related to the effects of the food recall measure. For example, 14 participants said they had eaten less (particularly fewer snacks) to make the measure easier to complete or because they became more aware of what they were eating. Five participants also reported trying to eat more healthily, again because the food recall measure made them more aware of what they were eating. Two participants said the recall measure meant they finished what was on their plate to avoid having to record leftovers and 2 participants said they had selected simpler foods that would be easier to record. 

## 4. Discussion

The results showed no effects of the mindful eating strategy on self-reported energy intake or on self-reported intake of saturated fat, added sugar, fiber or fruit/vegetables. This absence of effects was reinforced by the fact that there were also no consistent or significant correlations between these outcomes and the extent to which participants reported using the strategy. These findings are in line with a recent study by Tapper and Seguias [9] that examined the effects of mindful eating outside the laboratory over a half-day period and also failed to find effects on energy intake or intake of saturated fat, added sugar or fiber. In that study, the women who took part were older (mean age of 44 years), slightly heavier (mean BMI of 25.5) and 15% were dieting to lose weight. Taken together, these two studies suggest that instructing someone to focus on the sensory properties of their food will not necessarily improve their diet and that effects on intake found in the laboratory [3,4,5,6,7,8] will not necessarily generalize to effects outside the laboratory. A related study evaluating an 8-week “attentive eating” intervention with males and females with overweight/obesity reached similar conclusions when it failed to find any effects on weight loss or on food intake assessed over a 24-h period [19]. Thus, these findings question the utility of using brief advice to eat more mindfully as a general strategy for helping people limit their food intake.

However, an important difference between the current study and the laboratory studies that found significant effects may be the ways in which foods were served; the laboratory studies have tended to assess consumption of smaller pieces of food (such as biscuits broken into pieces) taken directly by participants from a larger serving. By contrast, outside the laboratory, servings may often be pre-measured, for example, a takeaway sandwich or a small bag of crisps. Thus, it is possible that this type of mindful eating is only effective where people find it hard to keep track of what they have eaten. As such, we may only see effects emerge in specific situations, for example, when eating from a buffet or helping oneself to food directly from a larger serving. Future research could explore this possibility by examining intake in settings where this type of eating may be more common, such as at family meals or social gatherings.

The results also showed no effects of the no distractions strategy on self-reported energy intake or on self-reported intake of saturated fat, added sugar, fiber or fruit/vegetables. However, in this case, exploratory analysis showed a trend toward a negative relationship between strategy use and calorie intake, consistent with other research that has shown that limiting distractions reduces intake [12,13,14,15]. This raises the possibility that a simple instruction to limit distractions whilst eating may help people reduce intake, but only where they are very motivated to do so. Indeed, comments made by participants indicated that this was a difficult strategy to implement and that in many cases participants were reluctant to change their habits. It is possible that motivation and adherence would be greater among those who wanted to reduce their energy intake in order to lose weight, especially if they believed that limiting distractions would help them achieve this goal. Nevertheless, it should be emphasized that the current negative association between strategy use and intake was not statistically significant so further research would be needed to substantiate these ideas.

It is important to note that there are a number of methodological limitations to the study that reduce the strength of the conclusions that can be drawn. First, there was no baseline dietary assessment, meaning that change in dietary intake could not be calculated, and tests of group differences assumed equal intake across groups at baseline. 

Second, habits can be difficult to change and participants’ ratings of strategy adherence showed a significant increase over the three days, suggesting that it took time for people to remember to use the strategies. With a longer study duration, there may have been further increases in adherence that may eventually have translated into significant effects. However, use of the audio was also low, so it is unclear to what extent participants were practicing the strategies as directed. Differences in adherence may also help explain the discrepancy with laboratory studies where adherence may have been higher. As such, future research could explore effects with participants who are more motivated to engage in the strategies and/or with a stronger intervention that includes, for example, in-person training with an instructor.

Additionally, it seems likely that the food recall measure reduced intake across all groups and/or that participants were under-reporting intake. Indeed, average daily energy needs for this sample, assuming seated work with no exercise, would be around 1791 kcal [20], substantially more than the average 1544 kcal reported in the current study. Under-reporting is a known problem with self-report measures of diet [21,22] and may limit the extent to which they are sensitive to relatively small changes in intake. This makes it difficult to collect accurate dietary intake data outside the laboratory setting. An alternative approach would be to examine changes in weight over time, but this would require a sustained intervention which would, in turn, need highly motivated participants. 

Finally, comments provided by participants also suggest that completing the food recall measure made them more aware of what they were eating which, in turn, prompted them to eat less or to eat more healthily. These comments are in line with other research that has shown effects of monitoring on intake [23]. Although the food recall measure would have suppressed intake equally across all three groups in the current study, it may have introduced floor effects whereby the no distraction and mindful eating strategies were no longer able to bring about any further increases in awareness/reductions in intake. However, as noted above, the absence of effects for the mindful eating strategy are consistent with Tapper and Seguias [9], where participants were unaware they would be asked to complete a food recall measure at the end of the day. 

Thus, these methodological limitations make it difficult to draw any firm conclusions about the effects of this type of mindful eating on intake. Nevertheless, it should also be noted that an absence of effects among this population would not rule out significant effects in other populations, for example, among those with a higher BMI. Establishing the mechanisms underlying laboratory effects could help researchers identify particular situations or populations where these strategies would be most likely to be beneficial. For example, if effects are largely driven by reductions in habitual eating, we would expect the strategies to align eating more closely with a person’s attitudes and motivations [2]. This would mean that the strategies would be most effective for those who were actively trying to lose weight or eat more healthily. If the mindful eating strategy works by slowing down the rate of eating, it may only be effective for a subset of people who are naturally fast eaters [24,25,26]. The possibility that the mindful eating strategy may be helpful for a minority of people is supported by some of the comments made by participants. For example, two individuals reported that the strategy had led them to seek food with more texture, something that has been shown to slow eating and reduce energy intake [27]. Increasing interest in personalized medicine makes it worth pursuing potential effects in population subgroups, especially given that mindful eating may have the additional benefit of increasing food enjoyment. 

## Figures and Tables

**Table 1:** Characteristics of study participants as a function of condition.

Characteristic	Condition		
	MIND (*n* = 33 ^a^)	CON-D (*n* = 33 ^b^)	CON-I (*n* = 33 ^b^)
Age in years (mean, SD)	21.21 (3.72)	21.47 (3.93)	24.48 (6.29)
BMI (mean, SD)	22.39 (1.35)	22.10 (1.20)	22.35 (1.56)
Asian/Asian British (%)	55%	36%	18%
Black/Black British (%)	3%	18%	9%
White (%)	33%	39%	67%
Use of computer for food recall on all days (%)	82%	85%	82%

^a^ *n* = 31 for BMI; ^b^ *n* = 30 for BMI.

**Table 2:** Mean (SD) daily energy intake (kcal) and daily intake of saturated fat, added sugar, fiber and fruit/vegetables (grams) for participants in each of the three conditions.

Outcome Measure	Condition		
	MIND (*n* = 33)	CON-D (*n* = 33)	CON-I (*n* = 33)
Energy	1594 (425)	1547 (614)	1540 (442)
Saturated fat	23 (8)	20 (12)	22 (10)
Added sugar	54 (33)	43 (41)	52 (36)
Fiber	12 (6)	14 (8)	12 (6)
Fruit and vegetables	215 (178)	212 (204)	168 (191)

**Table 3:** Mean (SD) ratings, on a scale of 1–5 (anchored by “not at all” and “very much”), of the extent to which MIND and CON-D participants attended to the sensory properties of their food/ate without distractions on each of the three study days.

Condition	Study Period			
	Day 1	Day 2	Day 3	Overall
MIND (*n* = 33)	3.70 (0.85)	3.70 (0.98)	4.24 (0.71)	3.88 (0.64)
CON-D (*n* = 33)	3.45 (0.91)	3.61 (0.83)	3.73 (0.98)	3.60 (0.62)

**Table 4:** Correlations between mean self-reported strategy use and mean macronutrient intake in the MIND and CON-D conditions.

Condition	Outcome Measure				
	Energy	Saturated fat	Added sugar	Fiber	Fruit/vegetables
MIND (*n* = 33)	−0.02	−0.10	0.10	0.04	0.04
CON-D (*n* = 33)	−0.31 *	−0.07	0	−0.13	−0.07

* *p* = 0.085.

**Table 5:** Percentage of participants in each of the three conditions reporting different levels of relative pleasure experienced from food during the study.

Relative Pleasure	Condition		
	MIND (*n* = 33)	CON-D (*n* = 33)	CON-I (*n* = 33)
Less than usual	0%	9%	9%
Same as usual	61%	64%	79%
More than usual	39%	27%	12%

**Table 6:** Percentage of participants who reported that the study influenced the amount of food they ate and the type of food they ate.

Variable Influenced	Condition		
	MIND (*n* = 33)	CON-D (*n* = 33)	CON-I (*n* = 33)
Amount of food	33%	27%	15%
Type of food	24%	15%	21%

## Data Availability

The data presented in this study are available on request from the corresponding author. The data are not publicly available due to the nature of consent provided by participants.

## Supplementary Materials

The following supporting information can be downloaded at: https://www.mdpi.com/article/10.3390/nu14051043/s1, Scripts for audio recordings; Messages.
