# Supplementary material for: A Randomized Controlled Trial Examining the Effects of Mindful Eating and Eating without Distractions on Food Intake over a Three-Day Period
Source: Nutrients. 2022 Feb 28;14(5):1043. doi: 10.3390/nu14051043 (PMC8912312; doi:10.3390/nu14051043)
Supplement: Supplementary file 1 [file nutrients-14-01043-s001.zip › S1.pdf]

Scripts for audio recordings

MIND group (attend to the sensory properties of food)

As you eat your food, imagine that you are seeing the food for the first time. Examine the food. Look at the colour and feel the texture of the food. Focus on its weight and temperature. Smell the food. Does it have a smell? Is it sweet smelling or sour? Take in the odor, whatever it is, if there is one. Then move the food over your lips, first the lower lip and then the upper. Feel the temperature and texture of the food as it moves over your lips. Does it feel warm or cool? Smooth or rough? As you then gently bite into the food, feel its shape, texture, and temperature. When you are ready, begin to chew the food, feeling its every aspect. Without swallowing yet, notice the bare sensations of taste and texture in the mouth and how these may change over time, moment by moment. Is the texture hard or soft? Crispy or chewy? How does this texture change as you chew the food? Also notice the sound the food makes as you chew. Is the sound loud or quiet? Sharp or muffled? How does the sound vary with each bite? Start to taste the bursting of flavor. What does the food taste like? Is it sweet? Is it salty? Does the flavor vary in different parts of your mouth? Is it similar to any other flavors you know? What different flavors can you detect? Now, work the food toward the back of the throat and swallow it, observing its path as it traverses the throat and finally enters the stomach. Try to think about these different qualities of the food as you eat it. As you eat your food, try to remember to focus on the look, feel, smell, taste, texture, and sound of each bite.

You may play this audio clip whilst eating to help remind you of these things.

CON-D group (eat without distractions)

As you eat your food, try to avoid all distractions. Try to eat alone in a quiet setting. You can be sitting or standing but try not to eat whilst walking or travelling. If others are with you, try to avoid having prolonged conversations. Don’t eat in front of the television and try to put your phone or laptop elsewhere to help you avoid the temptation to send messages, make calls, or check social media. Avoid reading magazines or books while you eat and try not to listen to music or the radio. Do not attempt to do any office or school related work. As you eat your food, try to remember to eat without any distractions.

You may play this audio clip just before eating to help remind you of these things.
