# Supplementary material for: A Randomized Controlled Trial Examining the Effects of Mindful Eating and Eating without Distractions on Food Intake over a Three-Day Period
Source: Nutrients. 2022 Feb 28;14(5):1043. doi: 10.3390/nu14051043 (PMC8912312; doi:10.3390/nu14051043)
Supplement: Supplementary file 1 [file nutrients-14-01043-s001.zip › S2.pdf]

Seguias and Tapper (2022)

Messages

M = MIND group (attend to the sensory properties of food)
C = CON-D group (eat without distractions)

Day1

M, 7am: Good morning! A little reminder to focus on your food whenever you eat today. If you can, try to listen to the audio clip whilst eating.

C, 7am: Good morning! A little reminder to try to avoid distractions whenever you eat today. If you can, try to listen to the audio clip just before eating.

M, 10am: Another reminder to focus on the sensory properties of your food when you eat today. Think about the texture. Is it hard or soft? Crispy or chewy?

C, 10am: Another reminder to avoid all types of distraction when you eat today. Are you somewhere you won't be disturbed? Have you switched off your phone?

M, 12pm: As you eat your lunch, try to look closely at the food's size, shape and colour. Notice the sound it makes as you bite into it.

C, 12pm: As you eat your lunch, try to ensure you are in a quiet setting. If others are with you, try to minimise conversation whilst eating.

M, 3pm: Don't forget to focus on the sensory properties of the food you eat today! Try to notice the flavour and texture of each bite. How do these change as you chew?

C, 3pm: Don't forget to eat without distractions today! Try to avoid conversations whilst eating. Have you turned off your phone?

M, 5:30pm: As you eat your evening meal, try to focus on the different qualities of each food, paying particular attention to their smell, taste and texture.

C, 5:30pm: As you eat your evening meal, try to avoid distractions, such as watching TV or reading.

Day 2:

M, 7am: Good morning! Please try to listen to the audio clip at least once whilst eating today and try to focus on the sensory properties of the food every time you eat.

C, 7am: Good morning! Please try to listen to the audio clip at least once before eating today and try to avoid distractions every time you eat.

M, 12pm: When you have lunch, take some time to notice the temperature and smell of the food.

C, 12pm: When you have lunch, take some time to set aside anything you're reading or working on.

M, 5:30pm While eating your evening meal, remember to focus on the different flavours and textures of each food.

C, 5:30pm While eating your evening meal, remember to avoid doing any household or school related tasks.

Day 3:

M, 10am: Don't forget to listen to the audio clip at least once today! And remember to focus on the sensory properties of the food every time you eat.

C, 10am: Don't forget to listen to the audio clip at least once today! And remember to avoid all distractions every time you eat.
